# Comparison of abusive head trauma *versus* non‐inflicted subdural haematoma in infants: A retrospective cohort study

**DOI:** 10.1111/1742-6723.14028

**Published:** 2022-06-04

**Authors:** Peter J Snelling, Anthony Aruljoe Thanasingam, Philip Jones, Jan Connors

**Affiliations:** ^1^ Emergency Department Gold Coast University Hospital Gold Coast Queensland Australia; ^2^ Child Protection Unit Mater Children's Hospital Brisbane Queensland Australia; ^3^ School of Medicine and Dentistry Griffith University Gold Coast Queensland Australia; ^4^ Child Health Research Centre The University of Queensland Brisbane Queensland Australia; ^5^ Emergency and Trauma Centre Royal Brisbane and Women's Hospital Brisbane Queensland Australia; ^6^ Child Protection and Forensic Medical Service Queensland Children's Hospital Brisbane Queensland Australia

**Keywords:** haematoma, infant, inflicted, non‐accidental, head injury, paediatric, child protection, abusive, subdural haemorrhage, trauma

## Abstract

**Objectives:**

To compare the characteristics of subdural haematoma (SDH) in children under the age of 2 years, between inflicted, otherwise known as abusive head trauma (AHT), and non‐inflicted aetiologies.

**Methods:**

This was a retrospective cohort study of 37 cases of SDH in children under the age of 2 years presenting to the ED at an Australian tertiary children's hospital between January 2009 and December 2012 and been assessed by the Child Protection Unit. SDH aetiology was classified into AHT and non‐inflicted groups, based on child protection interagency outcome. These groups were compared to determine clinical associations with AHT.

**Results:**

Of the 37 infants with SDH, 20 cases were deemed due to AHT, whereas 17 cases were determined to be non‐inflicted SDH (15 cases due to accidental trauma and two cases due to congenital benign enlargement of the subarachnoid space). SDH due to AHT was associated with antenatal maternal drug use, previous Department of Child Safety involvement, delayed presentation, history of seizures, floppiness or altered level of consciousness; extracranial findings of fractures, bruising or retinal haemorrhages; radiological findings of >5 mm depth, bilateral, inter‐hemispheric blood, posterior fossa blood and diffusion restriction; and outcomes of death or permanent disability. Non‐inflicted SDH was associated with witnessed injury, falls and scalp haematoma on imaging.

**Conclusions:**

Infant SDH due to AHT accounts for high mortality and morbidity. Early identification of these patients in the ED with referral to specialised units that investigate for potential child abuse is essential.

## Graphical abstract

This was a retrospective cohort study of 37 cases of subdural haematoma (SDH) in children under the age of 2 years presenting to the ED, who had been assessed by the Child Protection Unit at an Australian tertiary children's hospital, between January 2009 and December 2012. Infant SDH because of abusive head trauma accounts for high mortality and morbidity and should be considered in infants presenting to the ED with seizures, floppiness, apnoea or altered level of consciousness.

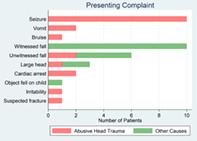

**Key findings**

- In this retrospective cohort study of 37 cases of SDH in infants less than 2 years of age presenting to the ED, 17 cases were due to AHT and 17 cases were non‐inflicted.
- Presenting features that should alert a clinician to AHT included seizures, floppiness, apnoea, and/or altered level of consciousness, whereas non‐inflicted SDH were associated with witnessed injury, falls and scalp haematoma on imaging.
- Medical evaluation of infants presenting with SDH should consist of a careful clinical assessment, medical imaging and eye examination to determine the underlying cause, as there is high mortality and morbidity associated with AHT.

## Introduction

Children frequently present to the ED with head injury. Injuries are usually mild, with most patients discharged home after clinical assessment and a period of observation.^1^ However, the risk of inflicted (non‐accidental) head injury, otherwise known as abusive head trauma (AHT), increases substantially for children under 2 years of age, accounting for greater than 80% of deaths due to head injury,^2^ and is closely related with the formation of subdural haematoma (SDH).^3^, ^4^, ^5^, ^6^, ^7^ Although the incidence of SDH (any cause) has been estimated at 12 cases per 100 000 for infants under 2 years of age,^8^, ^9^, ^10^, ^11^, ^12^, ^13^ given the high association of AHT in this age group, clinicians must maintain a high index of suspicion when assessing these children in the ED.

The main mechanism of SDH formation in infants from AHT is due to the tearing of the dural bridging veins from acceleration–deceleration and angular rotation forces applied to the skull and its contents, usually due to forceful shaking of the child, while being held around the trunk.^3^, ^4^, ^5^, ^6^, ^8^, ^14^ Infants are prone to SDH formation from this mechanism due to having a large head compared to the body, higher water content in the brain, poor cervical spine musculature, large subarachnoid space and immature myelination.^15^, ^16^ This type of AHT often results in a range of concomitant injuries, such as retinal haemorrhages, rib fractures and long bone metaphyseal fractures.^4^, ^6^ Direct impact to the head may also cause SDH, which may coincide with external signs of trauma at the site of impact.^3^, ^5^, ^8^, ^17^

Children with SDHs often present to the ED with non‐specific signs and symptoms, which can make them difficult to diagnose.^18^ These can include minor bruising, lethargy, reduced feeding, seizures or reduced level of consciousness.^19^, ^20^, ^21^ The diagnosis is made with neuroimaging, including US for infants with open fontanelles, CT and MRI, which are the most sensitive.^18^ Although SDH in young children should raise suspicion for AHT, it is imperative that other causes must be excluded. Non‐inflicted causes of SDH are extensive^22^ and include perinatal/birth‐related trauma, congenital brain condition (e.g. benign enlargement of the subarachnoid spaces), genetic (e.g. Ehlers–Danlos syndrome), accidental trauma, inborn errors of metabolism (e.g. glutaric aciduria type 1), infection (e.g. bacterial meningitis), coagulopathy (e.g. haemorrhagic disease of the newborn) or congenital vascular malformations.^3^, ^6^, ^7^, ^8^, ^9^, ^14^, ^17^, ^23^, ^24^, ^25^, ^26^

Therefore, a multi‐disciplinary approach is required, including specialist doctors, government statutory authority, and specialist police to diagnose, investigate and manage these children. The presence of SDH without plausible traumatic mechanism in the context of a child's development is highly suggestive of AHT and warrants full evaluation. This initial assessment is usually conducted by ED, with the involvement of the hospital's Child Protection Unit (CPU). The role of the CPU is to medically evaluate whether an injury is inflicted and warrants notification to the relevant statutory authority and relevant police service. In Queensland, the final determination of SDH being due to AHT is via interagency meetings involving representatives from the government statutory authority, CPU paediatrician, police service and department of education. Representatives share information under law and provide expert opinion on the evidence for the government's statutory authority to substantiate AHT.

The purpose of the present study was to compare the clinical characteristics, investigative features and outcomes of children under the age of 2 years diagnosed with SDH, between those established to be due to AHT and those due to other causes.

## Methods

### Study design and setting

This retrospective cohort study was conducted using data from the hospital medical records between January 2009 and December 2012 at the Mater Children's Hospital in Queensland, Australia. The Mater Children's Hospital was a tertiary paediatric (<16 years of age) centre that averaged approximately 45 000 ED presentations per annum during this time period. The Mater Health Services Human Research Ethics Committee approved the study (20133‐33).

### Selection of patients

Patients were eligible for the study if they were under the age of 2 years and diagnosed with SDH. Inclusion criteria included presenting undifferentiated to the ED and undergoing a comprehensive assessment by a CPU paediatrician. As it is a mandatory legal requirement in Australia to report any reasonable suspicion of child abuse and neglect, patients who were not referred to the CPU were excluded from the present study. A clinical coding search of the medical records was conducted using a combination of the following terms: ‘subdural’ and ‘haematoma’, ‘haemorrhage’, ‘collection’, ‘hygroma’ or ‘effusion’, including the International Classification of Diseases, 10th Revision discharge codes I620 and S065. These patient medical records were then compared against the hospital's internal CPU database, which records the outcomes of interagency meetings.

### Data collection

Data were manually extracted from the medical records by a reviewer blinded to the final SDH category in the CPU records. Pre‐determined categories included: demographics, presenting signs and symptoms, birth history, psychosocial background, investigations, clinical course and outcomes (death and disability up to 12 months from time of discharge). Data recording was standardised to ensure uniform handling of data that was ambiguous, missing or unknown. Socioeconomic status percentile for patient residential postcode was obtained from the Australian Bureau of Statistic Socioeconomic Indexes for Areas.^27^

### Outcome measures

The primary outcome of the study was the association of clinical and radiological features with AHT, compared to non‐inflicted aetiologies, in children under 2 years of age with SDH. SDH due to AHT was determined on the basis of interagency meeting outcome, with all other causes combined in a separate non‐inflicted SDH group.

### Data analysis

The association between group (AHT/other) and continuous outcomes was investigated using ANOVA (parametric data) or Wilcoxon rank‐sum test (non‐parametric data). The association between group and categorical outcomes was investigated using Fisher's exact test (two‐sided). Odds ratios (ORs) were presented for categorical outcomes, with 95% confidence intervals (CIs) calculated using the exact method. For variables with missing data, complete case analysis was performed, followed by sensitivity analysis, to evaluate statistically significant findings. Data were analysed using Stata/IC v14 (StataCorp, College Station, TX, USA).

## Results

Forty‐four infants with SDH were initially identified from the medical records across the 4‐year period using our search strategy. Four children with SDH as a direct result of neurosurgery or motor vehicle accident were not included as they did not present undifferentiated to the ED, with an additional three children excluded due to no recorded consultation with the CPU, leaving a total of 37 patients in the study.

Patient demographics, perinatal, social and medical history are outlined in Table 1. Twenty of these patients were determined to be AHT as per interagency investigation, with four having perpetrator confessions (one mother, one father and two mothers' partner). Of the 17 remaining SDH patients, 15 were determined to be a result of accidental trauma and two were due to an underlying congenital brain condition, known as benign enlargement of the subarachnoid space in infancy.^26^ There were no differences between groups with respect to median age, sex or residential postcode socioeconomic index. Antenatal recreational drug use (35% *vs* 0%) and previous Department of Child Safety involvement (30% *vs* 0%) were more common in the AHT group.

**TABLE 1:** Patient demographics by AHT *versus* other causes of SDH

	AHT (*n* = 20)	Non‐inflicted SDH (*n* = 17)	Odds ratio (95% CI)	*P*‐value
Demographics				
Age (months)	4.5 (2–10)	6 (3–11)	N/A	0.37
Male (sex)	10 (50)	11 (64)	0.54 (0.11, 2.4)	0.51
SES centile	48 (23)	49 (31)	N/A	0.90
Perinatal, social and medical history				
Multiple births	3 (15)	0 (0)	N/A	0.23
Prematurity	3 (15)	0 (0)	N/A	0.23
Antenatal drug use	7 (35)	0 (0)	N/A	0.009
Special care nursery	5 (25)	3 (18)	1.6 (0.24, 11.8)	0.70
Vitamin K administration†	16 (89)	17 (100)	0 (0, 2.0)	0.49
Maternal mental health	4 (20)	0 (0)	N/A	0.11
Previous Department of Child Safety involvement	6 (30)	0 (0)	N/A	0.02
Household violence	4 (20)	0 (0)	N/A	0.11
Household drug use	5 (25)	0 (0)	N/A	0.05
Vaccinations up‐to‐date†	13 (72)	13 (88)	0.35 (0.03, 2.6)	0.40
Developmental delay	3 (15)	1 (6)	2.8 (0.20, 160)	0.61

† Data missing for Vitamin K administration and vaccinations up‐to‐date in two patients in the AHT group.

Results shown as number (% of subgroup), median (interquartile range) or mean (±standard deviation). AHT, abusive head trauma; CI, confidence interval; SDH, subdural haematoma; SES centile, socioeconomic status percentile of patient residential postcode.

**Figure 1:**
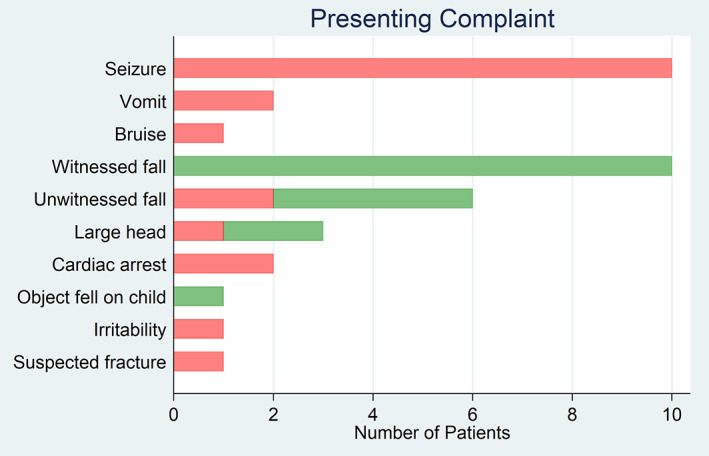
Number of patients (*x*‐axis) for each presenting complaint (*y*‐axis) for abusive head trauma and non‐inflicted subdural haematoma. (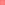), abusive head trauma and (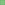), non‐inflicted subdural haematoma.

The most common presenting complaint for patients with AHT was seizure (*n* = 10), compared to a fall in patients with non‐inflicted SDH (*n* = 14; 10 witnessed, four unwitnessed; Fig. 1). Delay greater than 24 h in presentation (55% *vs* 6%, OR 20, 95% CI 2.0–890), altered level of consciousness (50% *vs* 12%, OR 7.5, 95% CI 1.2–80), floppiness (40.0% *vs* 0%), poor feeding (35% *vs* 0%) and apnoea (35% *vs* 0%) were associated with AHT. A reported history of injury (94% *vs* 35%, OR 0.03, 95% CI 0.001–0.33) and a history of a fall (88% *vs* 25%, OR 0.04, 95% CI 0.004–0.32) were associated with non‐inflicted SDH. Table 2 summarises these historical features.

**TABLE 2:** Presenting signs and symptoms, history of injury and other historical features by AHT *versus* other causes of SDH

	AHT (*n* = 20)	Non‐inflicted SDH (*n* = 17)	Odds ratio (95% CI)	*P*‐value
History of injury				
Reported history of injury	7 (35)	16 (94)	0.03 (0.00, 0.33)	<0.001
Witnessed injury	6 (30)	11 (65)	0.23 (0.05, 1.1)	0.05
Reported sibling caused injury	3 (15)	1 (6)	2.8 (0.20, 160)	0.61
Known presentation delay	11 (55)	1 (6)	20 (2.0, 890)	0.002
Fall	5 (25)	15 (88)	0.04 (0.004, 0.32)	<0.001
Fall over 1 m height	1 (5)	6 (35)	0.09 (0.002, 1.0)	0.03
History of shaking	2 (10)	0 (0)	N/A	0.49
History of burn	1 (5)	0 (0)	N/A	1.0
Historical findings				
Floppiness	8 (40)	0 (0)	N/A	0.004
Altered level of consciousness	10 (50)	2 (12)	7.5 (1.2, 80)	0.02
Collapse	5 (25)	0 (0)	N/A	0.05
Vomiting	8 (40)	6 (35)	1.2 (0.27, 5.8)	1.0
Irritability	4 (20)	1 (6)	4.0 (0.33, 210)	0.35
Lethargy	6 (30)	2 (12)	3.2 (0.46, 37)	0.25
Seizure	12 (60)	2 (12)	11 (1.7, 120)	0.006
Poor feeding	7 (35)	0 (0)	N/A	0.009
Enlarging head circumference	1 (5)	2 (12)	0.39 (0.006, 8.5)	0.58
Apnoea	7 (35)	0 (0)	N/A	0.009
Diarrhoea	3 (15)	0 (0)	N/A	0.23
Pallor	4 (20)	0 (0)	N/A	0.11
Fever	2 (10)	0 (0)	N/A	0.49
Posturing	5 (35)	2 (12)	2.5 (0.33, 29)	0.42

Results shown as number (% of subgroup). AHT, abusive head trauma; CI, confidence interval; SDH, subdural haematoma.

Bruising of any location was associated with AHT, whereas scalp haematoma was associated with the non‐inflicted group. Only 25 patients underwent fundoscopy, with all patients with retinal haemorrhages being detected in the AHT group (14/19 *vs* 0/5). However, expert fundoscopy was not performed in 12 patients in the non‐inflicted SDH group and one patient in the AHT group. Skeletal surveys were conducted in all cases of AHT, with rib and metaphyseal fractures showing associations with AHT. Extracranial fractures were only seen in the AHT group, whereas skull fractures were associated with the non‐inflicted group. In terms of neuroradiological findings, having both SDH and effusion, bilateral SDH, SDH depth >5 mm, inter‐hemispheric blood, posterior fossa blood and diffusion restriction were all associated with AHT, whereas scalp haematoma (e.g. subgaleal) and small, focal SDH beneath a simple, linear skull fracture were associated with the non‐inflicted group. Examination and radiological findings are described in Table 3.

**TABLE 3:** Diagnosed injuries by AHT *versus* other causes of SDH

	AHT (*n* = 20)	Non‐inflicted SDH (*n* = 17)	Odds ratio (95% CI)	*P*‐value
Examination findings				
Bruising	16 (80)	3 (13)	19 (2.9, 140)	<0.001
Laceration	1 (5)	0 (0)	N/A	1.0
Abrasion	2 (10)	0 (0)	N/A	0.49
Burn	2 (10)	0 (0)	N/A	0.49
Scalp haematoma	1 (5)	13 (76)	0.02 (0.001, 0.18)	<0.001
Retinal haemorrhages†	14 (74)	0 (0)	N/A	0.006
Neuroradiological findings				
Bilateral injuries	15 (75)	5 (29)	7.2 (1.4, 40)	0.009
More than two injuries	5 (25)	0 (0)	N/A	0.05
Haemorrhage and effusion	8 (40)	0 (0)	N/A	0.004
Depth (>5 mm)	13 (65)	1 (6)	30 (3.0, 1300)	<0.001
Inter‐hemispheric blood	6 (30)	0 (0)	N/A	0.02
Posterior fossa blood	6 (30)	0 (0)	N/A	0.02
Midline shift	5 (25)	1 (6)	5.3 (0.49, 270)	0.19
Diffusion restriction	11 (55)	3 (18)	5.7 (1.0, 39)	0.04
Parenchymal injury	5 (25)	2 (12)	2.5 (0.33, 29)	0.42
Cerebral oedema	5 (25)	1 (6)	5.3 (0.49, 270)	0.19
Diffuse axonal injury	4 (20)	0 (0)	N/A	0.11
Subarachnoid haemorrhage	6 (30)	4 (24)	1.4 (0.25, 8.3)	0.73
Scalp haematoma	0 (0)	4 (24)	0 (0, 0.70)	0.04
Small, focal SDH beneath simple, linear skull fracture	1 (5)	12 (71)	0.02 (<0.001, 0.24)	<0.001
Other radiological findings				
Skull fracture	4 (20)	12 (71)	0.10 (0.02, 0.58)	0.003
Facial fracture	1 (5)	0 (0)	N/A	1.0
Metaphyseal fracture	6 (30)	0 (0)	N/A	0.02
Rib fracture	6 (30)	0 (0)	N/A	0.02
Long bone fracture	4 (20)	0 (0)	N/A	0.11
Vertebral fracture	1 (5)	0 (0)	N/A	1.0

† Fundoscopy of both retinae not performed in one patient in the AHT and 12 patients in the non‐inflicted SDH group.

Results shown as number (% of subgroup). AHT, abusive head trauma; CI, confidence interval; SDH, subdural haematoma.

Treatment and outcome data are summarised in Table 4. The AHT group had a longer median length of stay of 14 days (interquartile range [IQR] 7.5–22.5 days), compared to 4 days (IQR 4–5 days) in the non‐inflicted SDH group. Admission to the paediatric intensive care unit (PICU) was required for 17 infants, with 14 of these from the AHT group (70% *vs* 18%, OR 11, 95% CI 1.9–76). Neurosurgery was required in five patients with AHT, who underwent SDH decompression and insertion of subdural peritoneal shunt, whereas one patient in the non‐inflicted group underwent craniotomy solely for skull fragment elevation. Three infants (15%) with AHT died, with no deaths occurring in the non‐inflicted SDH group. Disability was only seen in the AHT group, with 11 having permanent neurological disability, including developmental delay, spasticity or epilepsy, and eight having permanent visual impairment. These results remained statistically significant after sensitivity analysis for missing outcome data (Table 4).

**TABLE 4:** Treatment and outcomes by AHT *versus* other causes of SDH

	AHT (*n* = 20)†	Non‐inflicted SDH (*n* = 17)	Odds ratio (95% CI)	*P*‐value
Treatment				
Antiepileptic drug therapy	15 (75)	2 (12)	22.5 (3.1, 240)	<0.001
PICU	14 (70)	3 (18)	11 (1.9, 76)	0.003
Surgery	5 (25)	1 (6)	5.3 (0.49, 270)	0.19
Outcome				
Length of stay (days)	14 (7.5–22.5)	4 (4–5)	N/A	<0.001
Death	3 (15)	0 (0)	N/A	0.23
Visual impairment	8† (57)	0 (0)	N/A	<0.001
Developmental delay	11† (69)	0 (0)	N/A	<0.001
Neurological impairment/epilepsy	11† (69)	0 (0)	N/A	<0.001
Dept child safety intervention	20 (100)	0 (0)	N/A	<0.001

† Outcome data missing for visual impairment in four patients, developmental delay in two patients and neurological impairment/epilepsy in two patients, all in AHT group. Two patients who died were not included in analysis. Findings remain statistically significant if all patients with missing data assumed to have no disability (Visual impairment: *P* = 0.003. Developmental delay: *P* < 0.001. Neurological impairment/epilepsy: *P* < 0.001). Results shown as number (% of subgroup) or median (interquartile range). AHT, abusive head trauma; CI, confidence interval; SDH, subdural haematoma.

## Discussion

In this Australian retrospective cohort study, a total of 37 infants with SDH were identified, with 20 patients categorised in the AHT group. Although three patients were excluded from the study due to no record of CPU involvement, it is highly unlikely that any of these represented AHT due to low level of concern, with either non‐referral or that the discussion with CPU was not recorded. Overall, traumatic SDH accounted for the majority (95%) of cases, with two cases of SDH due to the underlying congenital condition of benign enlargement of subarachnoid space in infancy, known to cause subdural effusion and macrocephaly.^26^ No SDH in the present study was found to be due to birth trauma, coagulopathy or metabolic disease, confirming the rarity of these conditions.^6^, ^7^, ^8^, ^9^

Both SDH groups had similar demographics, including age, sex and residential postcode socioeconomic status. Male sex has been associated with AHT,^9^, ^11^, ^12^, ^28^, ^29^ previously cited to be a risk factor for cultural reasons and intolerance of male crying,^4^ but this was not reflected in our study or the one by Feldman *et al*.^6^ Low socioeconomic status is also typically reported to be associated with AHT,^4^ but this trend was not replicated in our study, albeit when the socioeconomic status was generalised across households within a postcode.

The AHT group was associated with longer duration of admissions and disability, which reflects being generally of a more severe nature. This is further supported by the association of SDH due to AHT with seizures, floppiness, altered level of consciousness and apnoea, which are features of the traumatic encephalopathy that ensues from repeated angular rotation and acceleration–deceleration forces. Other studies corroborate the association of seizures or apnoea at time of presentation.^4^, ^6^, ^7^, ^10^, ^29^, ^30^ Delayed presentation greater than 24 h from time of injury was another significant difference between groups, which logically could be related to perpetrators of AHT attempting to conceal inflicted injuries.^7^ Consequently, infants often present in extremis or cardiac arrest.^4^, ^7^, ^9^, ^29^

Logically, the non‐inflicted SDH group was associated with a history of a witnessed fall or with provision of a suitable mechanism of injury, in keeping with previous literature.^3^, ^4^, ^5^, ^6^, ^7^, ^9^, ^10^, ^17^ Clinical features and history of the mechanism are explored in detail for consideration of whether a complete workup is required by the CPU. However, given the potential for perpetrators of AHT to concoct plausible stories, these features are not entirely reassuring. The association between AHT and social risk factors, including maternal drug use in pregnancy and previous involvement with the Department of Child Safety, is in keeping with what has been reported in literature.^4^, ^6^, ^9^, ^10^, ^11^ These social risk factors warrant further psychosocial review by a social worker and involvement of a CPU paediatrician, but should not be considered pathognomonic for AHT.

Extracranial injuries strongly associated with SDH due to AHT in our study included retinal haemorrhages, metaphyseal fractures, rib fractures and facial or abdominal bruising, in keeping with previous literature.^3^, ^4^, ^5^, ^6^, ^7^, ^9^, ^10^, ^17^ These features reflect a more severe mechanism of injury, in conjunction with more severe intracranial injuries, which aids the establishment of AHT. However, the pattern and severity are important, as, for example, focal retinal haemorrhages can occur in the setting of defined trauma.^9^, ^12^, ^29^ Conversely, the finding of a scalp haematoma in conjunction with a mechanism, such as a simple fall, was strongly associated with accidental trauma. However, this is not a specific finding, given that AHT can also occur with direct impact injuries.^15^, ^18^

MRI is reported to be more sensitive than CT for the detection of SDH and other associated intracranial injuries in AHT.^4^, ^10^ All AHT in our study received neuroimaging, with all undergoing MRI apart from a single infant with AHT who had small volume SDH and extracranial features suggestive of inflicted injuries, including bruising and fractures. Other neuroimaging findings in our study favouring the likelihood of SDH due to AHT, including cerebral ischaemia (diffusion restriction), cerebral oedema and SDH of various ages and multiple sites, in keeping with literature.^3^, ^4^, ^5^, ^6^, ^7^, ^9^, ^11^, ^17^ Alternatively, scalp haematoma on MRI was strongly associated with non‐inflicted head trauma, consistent with a mechanism of blunt force trauma, such as a simple fall against a hard surface.

Admission to PICU was statistically significant for AHT, which is in keeping with a higher severity of injury. Treatment with antiepileptics was also associated with AHT, which soundly follows the higher rate of seizures seen in this group in conjunction with traumatic encephalopathy or trend towards larger SDH. Prophylactic antiepileptic treatment was also provided for severe head injuries without the development of seizures. A higher proportion of AHT underwent surgical intervention, again, most likely reflecting a higher severity of injury, similar to other studies.^4^, ^9^, ^11^, ^23^

Outcomes following AHT in infancy are poor, with the literature reporting mortality ranging from 5% to 36%,^4^, ^6^, ^9^, ^11^, ^12^, ^18^, ^28^ and morbidity in surviving children ranging from mild learning difficulties to severe physical and cognitive impairment.^4^, ^9^, ^10^, ^11^, ^12^, ^14^ Our study was no exception, with 15% mortality and 55% permanent neurological disability in the AHT group. No death or disability was reported for any of the non‐inflicted SDH group, but this has been reported to occur.^6^, ^7^, ^28^ Although the radiological finding of ischaemia or cerebral oedema has been reported to be associated with a poor neurological outcome,^4^ our study numbers were too small for analysis.

Our study had further similarities and differences to other reported studies of infant SDH. The majority of studies, like ours, used a retrospective design,^4^, ^7^, ^8^, ^11^, ^12^, ^13^, ^23^, ^28^, ^29^ with only two prospective studies.^6^, ^9^ The majority reported children under 2 years with undifferentiated SDH,^7^, ^11^, ^12^, ^13^ whereas others included older children,^4^, ^6^, ^23^, ^28^ head injuries more broadly,^4^, ^28^, ^29^ or AHT alone.^4^ Many studies also excluded patients with SDH due to known causes, such as neurosurgery.^6^, ^11^, ^23^ In terms of AHT determination, the majority used a combination of legal and medical child protection outcomes,^4^, ^8^, ^9^, ^11^, ^12^, ^23^, ^28^, ^29^ whereas others used the outcome from the hospital child protection team alone.^6^, ^7^, ^13^ A high proportion of SDH were caused by AHT in the two largest studies.^9^, ^29^

The limitations of the present study include its retrospective design, no second reviewer for data extraction inter‐rater reliability, and small numbers for statistical analysis. A broader range of SDH cases would likely to have been included if data were collected over a longer period. As many variables were evaluated for association with AHT, robust correction for multiple comparisons was not feasible, significantly increasing the likelihood of one or more Type I errors. Another limitation was the risk of incorporation bias, as the variables of interest include established risk factors for AHT that would have been considered in determining this finding. This likely leads to an overestimation of the association between this outcome and the variables of interest. However, this was mitigated by the determination of AHT following a comprehensive CPU assessment, interagency meeting, and legal outcomes, which formulates an appropriate gold standard in keeping with other literature.^4^, ^8^, ^9^, ^11^, ^12^, ^23^, ^28^, ^29^ Additionally, the findings associated with AHT and non‐inflicted SDH in the present study were internally consistent and broadly in keeping with previous literature.

## Conclusion

Infant SDH due to AHT accounts for high mortality and morbidity, which makes it vital for early identification of these patients in the ED with referral to specialist units that investigate for potential child abuse. The medical evaluation of infants presenting with SDH should include a detailed review of potential accidental causes, exclusion of underlying medical conditions, and delineation of injuries with careful skin and oral examination, neuroimaging, skeletal survey and ophthalmologist retinal examination, to provide medical evidence for inter‐agency consideration. Features that should increase concern for SDH due to AHT include antenatal drug use, a history of floppiness, seizures, altered level of consciousness or apnoea, extracranial injuries such as facial or abdominal bruising, metaphyseal or rib fractures, and numerous radiological findings, such as bilateral SDH and/or >5 mm depth. Although this retrospective study provides further insights into the differentiation of SDH due to AHT *versus* other causes, there is considerable overlap of characteristics between groups and so a careful and thorough approach is required.

## Data Availability

No further data available.
